# The Use of Transvenous Lead Extraction of Non-Infected Leads to Prevent Long-Term Lead-Related Complications

**DOI:** 10.17691/stm2021.13.1.08

**Published:** 2021-02-28

**Authors:** S.A. Ayvazyan, A.B. Gamzaev, A.A. Palagina, K.G. Gorshenin, S.I. Buslaeva, A.A. Seregin, N.S. Konovalov, O.V. Sapelnikov

**Affiliations:** Cardiovascular Surgeon, Volga District Medical Centre of Federal Medical Biological Agency of Russia, 2 Nizhne-Volzhskaya naberezhnaya, Nizhny Novgorod, 603001, Russia; Professor, Department of X-ray Surgical Methods of Diagnosis and Treatment, Privolzhsky Research Medical University, 10/1 Minin and Pozharsky Square, Nizhny Novgorod, 603005, Russia; Resident, Department of X-ray Endovascular Diagnostics and Treatment, Privolzhsky Research Medical University, 10/1 Minin and Pozharsky Square, Nizhny Novgorod, 603005, Russia; Cardiologist, Volga District Medical Centre of Federal Medical Biological Agency of Russia, 2 Nizhne-Volzhskaya naberezhnaya, Nizhny Novgorod, 603001, Russia; Cardiologist, Volga District Medical Centre of Federal Medical Biological Agency of Russia, 2 Nizhne-Volzhskaya naberezhnaya, Nizhny Novgorod, 603001, Russia; Endovascular Surgeon, Head of the Department of X-ray Surgical Methods of Diagnosis and Treatment, Volga District Medical Centre of Federal Medical Biological Agency of Russia, 2 Nizhne-Volzhskaya naberezhnaya, Nizhny Novgorod, 603001, Russia; Resident, Department of X-ray Surgical Methods of Diagnosis and Treatment, Privolzhsky Research Medical University, 10/1 Minin and Pozharsky Square, Nizhny Novgorod, 603005, Russia; Cardiovascular Surgeon, Department of Cardiovascular Surgery, National Medical Research Center of Cardiology, 15a, 3^rd^ Cherepkovskaya St., Moscow, 121552, Russia

**Keywords:** transvenous lead extraction, non-infected superfluous lead, thoracoscopy, venous occlusion, cardiac implantable device, superior vena cava

## Abstract

**The aim of the investigation** was to study the issue of making challenging decisions concerning abandonment or removal of non-infected superfluous leads during lead revisions or cardiac implantable electronic device upgrades.

**Materials and Methods.:**

From 2010 to 2019, a total of 482 patients who had undergone cardiac implantable electronic device implantation in the past were admitted to hospital for generator replacement or lead revision. In 126 patients, 155 malfunctioning leads were found. Mean age of the patients was 59.2±16.7. Total venous occlusion was found in 10 cases of these patients. All patients were divided into two groups: extracted leads group (n=83) and abandoned leads group (n=43). The main factor which influenced our strategy was the mean age of the lead. In group 1 the mean age of the lead was 6.9±5.6 years. In group 2 it was about 12 years.

**Results.:**

Lead extraction was performed by manual traction in 69 (61.7%) leads, by lead locking device in 32 (28.5%) leads, and 11 (9.8%) leads were removed using TightRail rotating dilator sheath. In 1 case of total occlusion of the superior vena cava, we performed a video-assisted thoracoscopic lead extraction at the time of vein occlusion recanalisation and electronic device reimplantation. In abandoned leads group 3 patients had lead-related complications.

**Conclusion.:**

Transvenous lead extraction with the mean age of the lead less than 10 years is an effective and safe strategy. Preventive transvenous lead extraction of non-infected leads allows avoiding lead-related complications in the long-term period.

## Introduction

The use of cardiac implantable electronic devices (CIEDs) has been steadily increasing over the last years. CIEDs have become established as an important therapeutic modality of cardiovascular care for the treatment of patients with bradycardia, tachycardia, and heart failure. Lead management and extraction are increasingly essential components of the comprehensive care of patients with CIEDs [1]. In Russia, the number of cardiac pacemaker (PM) implantations, including biventricular ones, increased since 2006 to 2016 from 15,405 to 37,457, the number of cardioverter-defibrillators increased from 165 to 1418 [2].

Factors influencing the increased frequency include a recent rapid rise in de novo CIED implantations and frequent system revisions or upgrades and lead-related problems such as CIED infections, lead failures, and lead safety alerts [3–5]. The use of magnetic resonance imaging (MRI) has also grown. It is now predicted that 3 of 4 patients with CIEDs will need MRI once in their lifetime. It necessitates replacing such patients’ devices with MRI-resistance ones [6]. In 10–26% of patients with abandoned leads occlusion of upper extremity veins developed, which made it difficult to replace the leads [7–11].

The latest expert consensus statement on CIED lead management has mentioned that whether to abandon or remove those non-infected leads should be decided according to the clinical goal that balances both the risk of a lead removal and abandonment [12–15]. Abandoned leads are highly associated with several risks of complications, such as infections, arrhythmias, vascular trouble, tricuspid valve damage, and thromboembolism [1].

There are a number of lead extraction techniques. A few years after implantation, most leads can be removed by simple traction. If extraction cannot be performed in this way, a locking stylet can be used. This is a device that is placed into the lumen of the lead, opens in it, thereby preventing the current-conducting spiral from stretching during traction. If the locking stylet is ineffective, the next step is using mechanical dilator sheaths to disrupt and dilate the encapsulating fibrotic tissue fixing the lead. However, the use of these devices is associated with an increasing risk of complications. The most effective lead extraction device is the excimer laser [1, 16–19].

Transvenous lead extraction (TLE) has been established as a safe and effective method of lead removal with a high rate of procedural success and a low level of major complications. If it is not possible to use TLE, a hybrid approach has already proven its efficacy and safety [20–23].

Video-assisted thoracoscopic endocardial lead extraction allows the surgeon to remove the entire cardiac implantable electronic system at the time of vein occlusion recanalization and electronic device reimplantation without a sternotomy. This method allows preventing the development of various life-threatening complications, such as superior vena cava rupture with the mortality rate of 50%, and reducing complication rates in the postoperative period.

**The aim of the investigation** was to study the issue of making challenging decisions concerning abandonment or removal of non-infected superfluous leads during lead revisions or cardiac implantable electronic device upgrades.

## Materials and Methods

A total of 482 patients underwent the system upgrade procedure revisions at Volga District Medical Centre of Federal Medical Biological Agency of Russia (Nizhny Novgorod, Russia) from 2010 to 2019. 126 patients had dysfunctional leads (n=155). The patients’ age was 59.2±16.7 years. All patients were divided into two groups based on the treatment strategy. Group 1 (n=83) included patients who underwent TLE. Group 2 (n=43) consisted of patients with abandonment leads.

The main factor which influenced our strategy was the mean age of the lead. We did not extract superfluous leads over 15 years old. Contraindications to lead extraction were left ventricular ejection fraction less than 35% and patients’ age over 75 years. The risk of TLE (damage to the superior vena cava and cardiac chambers) in such patients is higher than lead-related complications in the long-term period. All TLE procedures were performed by experienced operators under conscious sedation or general anesthesia in the cardiac electrophysiology laboratory with immediate onsite cardiothoracic surgical cover. During the operations we carried out control using intracardiac Echo.

The study complies with the Declaration of Helsinki (2013) and was performed following approval by the Ethics Committee of Volga District Medical Centre of Federal Medical Biological Agency of Russia. Written informed consent was obtained from every patient.

In group 1, all leads were extracted transvenously through a subclavian using the following techniques: simple manual traction, using locking stylets, and a mechanical rotating dilator sheath. All patients underwent transthoracic echocardiography post-TLE (unless intraoperative transesophageal echocardiography was used) to assess for a hemodynamically significant pericardial effusion and valve damage as well as a chest radiograph. In 1 case of total occlusion of the superior vena cava, we performed a video-assisted thoracoscopic lead extraction at the time of vein occlusion recanalisation and electronic device reimplantation. In group 2, abandoned leads were sealed with a silicone cap.

Intraoperative complications and long-term complications were assessed in the study groups during the period from 3 months to 7 years.

## Results

**Figure 1:**
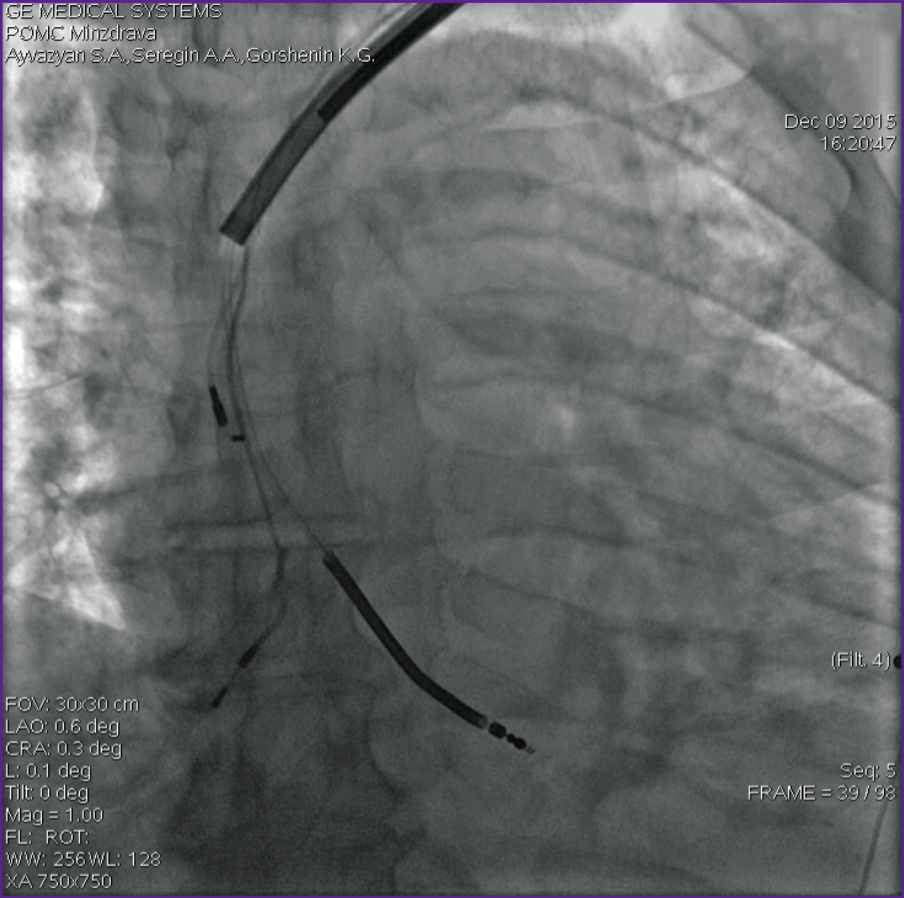
The use of the TightRail rotating dilator sheath (Spectranetics, Colorado Springs, CO, USA)

There were extracted 112 leads in group 1, of which 37 (33.7%) were atrial, 68 (60.7%) — right ventricular, 4 (3.5%) — dual coil leads, 3 (2.8%) — left ventricular. The number of leads extracted by simple manual traction was 69 (61.7%), 32 (28.5%) leads were extracted by traction using a locking stylet, and 11 (9.8%) leads were removed with the TightRail rotating dilator sheath (Spectranetics, Colorado Springs, CO, USA) (Figure 1).

The mean age of the lead was 6.9±5.6 years. Most of the leads were with active fixation. The success of the procedure was achieved in 98% of cases. During the TLE, normally functioning leads were damaged in 2 patients (they were extracted as well). There were no complications or deaths. Occlusion of the access vein (the subclavian vein, the innominate vein, the superior vena cava) was found in 10 patients (8%). Successful recanalization and lead re-implantation were performed in 4 patients. In two cases, recanalization was performed with a hydrophilic sheath, in two other cases, a rotating dilator sheath was used.

**Figure 2:**
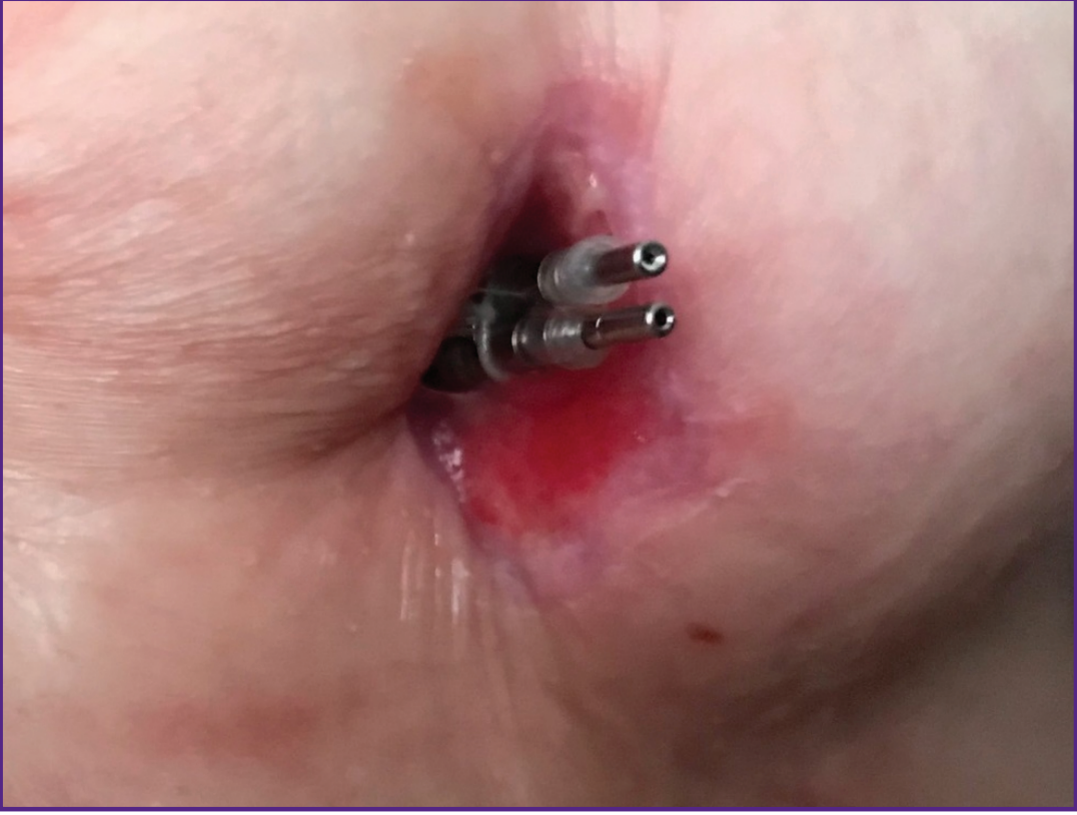
Skin erosion

In group 2, 3 patients with abandoned leads had lead-related complications in the period from 3 months to 7 years. In 2 cases lead-associated skin erosion developed (Figure 2). In these patients, the leads were removed using a rotating dilator sheath. In 1 case we observed endocarditis of the tricuspid valve. The patient undergoing tricuspid valve replacement and unfortunately the patient died in 3 month after the operation (sudden cardiac death).

## Conclusion

Transvenous lead extraction with the mean age of the lead less than 10–12 years is a safe and effective method of lead removal with a high rate of procedural success and a low level of major complications. Video-assisted thoracoscopic approach allowed us to control superior vena cava and provide the safety of the operation. The choice of a particular strategy in transvenous lead extraction depends on the risk factors connected with lead (mean age, type of fixation, the model of the lead), patient (age, left ventricular ejection fraction, respiratory and kidney insufficiency, sternotomy in patient’s history), operator experience with a specific technique.

Preventive transvenous lead extraction of non-infected leads allows avoiding lead-related complications in the long-term period.

## References

[1] Sood N., Martin D.T., Lampert R., Curtis J.P., Parzynski C., Clancy J (2018). Incidence and predictors of perioperative complications with transvenous lead extractions: real-world experience with National Cardiovascular Data Registry.. Circ Arrhythm Electrophysiol.

[2] Hindricks G., Camm J., Merkely B., Raatikainen P., Arnar D.O. (2017). The EHRA white book..

[3] Kusumoto F.M., Schoenfeld M.H., Wilkoff B.L., Berul C.I., Birgersdotter-Green U.M., Carrillo R., Cha Y.M., Clancy J., Deharo J.C., Ellenbogen K.A., Exner D., Hussein A.A., Kennergren C., Krahn A., Lee R., Love C.J., Madden R.A., Mazzetti H.A., Moore J.C., Parsonnet J., Patton K.K., Rozner M.A., Selzman K.A., Shoda M., Srivathsan K., Strathmore N.F., Swerdlow C.D., Tompkins C., Wazni O. (2017). HRS expert consensus statement on cardiovascular implantable electronic device lead management and extraction.. Heart Rhythm.

[4] Wilkoff B.L., Love C.J., Byrd C.L., Bongiorni M.G., Carrillo R.G., Crossley 3rd G.H, Epstein L.M., Friedman R.A., Kennergren C.E., Mitkowski P., Schaerf R.H., Wazni O.M. (2009). Heart Rhythm Society; American Heart Association. Transvenous lead extraction: Heart Rhythm Society expert consensus on facilities, training, indications, and patient management: this document was endorsed by the American Heart Association (AHA). Heart Rhythm.

[5] Halperin J.L., Levine G.N., Al-Khatib S.M., Birtcher K.K., Bozkurt B., Brindis R.G., Cigarroa J.E., Curtis L.H., Fleisher L.A., Gentile F., Gidding S., Hlatky M.A., Ikonomidis J., Joglar J., Pressler S.J., Wijeysundera D.N (2016). Further evolution of the ACC/AHA clinical practice guideline recommendation classification system: a report of the American College of Cardiology/American Heart Association Task Force on clinical practice guidelines.. J Am Coll Cardiol.

[6] Padmanabhan D., Kella D.K., Mehta R., Kapa S., Deshmukh A., Mulpuru S., Jaffe A.S., Felmlee J.P., Jondal M.L., Dalzell C.M., Asirvatham S.J., Cha Y.M., Watson Jr., R.E, Friedman P.A. (2018). Safety of magnetic resonance imaging in patients with legacy pacemakers and defibrillators and abandoned leads. Heart Rhythm.

[7] Ayvazyan S.A., Sharabrin E.G., Palagina A.A., Gorshenin K.G., Buslaeva S.I., Seregin A.A (2019). Recanalization of veins occlusion in patients after implantation of antiarrhythmic devices.. Diagnosticeskaa i intervencionnaa radiologia.

[8] Abu-El-Haija B., Bhave P.D., Campbell D.N., Mazur A., Hodgson-Zingman D.M., Cotarlan V., Giudici M.C. (2015). Venous stenosis after transvenous lead placement: a study of outcomes and risk factors in 212 consecutive patients. J Am Heart Assoc.

[9] Stoney W.S., Addlestone R.B., Alford Jr. W.C, Burrus G.R., Frist R.A., Thomas Jr. C.S. (1976). The incidence of venous thrombosis following long-term transvenous pacing. Ann Thorac Surg.

[10] Marcial J.M., Worley S.J (2018). Venous system interventions for device implantation.. Card Electrophysiol Clin.

[11] Li X., Ze F., Wang L., Li D., Duan J., Guo F., Yuan C., Li Y., Guo J (2014). Prevalence of venous occlusion in patients referred for lead extraction: implications for tool selection.. Europace.

[12] Higuchi S., Shoda M., Saito S., Kanai M., Kataoka S., Yazaki K., Yagishita D., Ejima K., Hagiwara N (2019). Safety and efficacy of transvenous lead extractions for noninfectious superfluous leads in a Japanese population: a single-center experience.. Pacing Clin Electrophysiol.

[13] Deshmukh A., Patel N., Noseworthy P. A., Patel A.A., Patel N., Arora S., Kapa S., Noheria A., Mulpuru S., Badheka A., Fischer A., Coffey J.O., Cha Y.M., Friedman P., Asirvatham S., Viles-Gonzalez J.F (2015). Trends in use and adverse outcomes associated with transvenous lead removal in the United States.. Circulation.

[14] Zucchelli G., Di Cori A., Segreti L., Laroche C., Blomstrom-Lundqvist C., Kutarski A., Regoli F., Butter C., Defaye P., Pasquié J.L., Auricchio A., Maggioni A.P., Bongiorni M.G. (2019). ELECTRa Investigators. Major cardiac and vascular complications after transvenous lead extraction: acute outcome and predictive factors from the ESC-EHRA ELECTRa (European Lead Extraction ConTRolled) registry. Europace.

[15] Sapelnikov O.V., Kulikov A.A., Cherkashin D.I., Grishin I.R., Nikolaeva O.A., Ardus D.F., Shiryaev A.A., Akchurin R.S (2019). Removal of electrodes of implanted systems. The state of the problem. Patologiya krovoobrashcheniya i kardiokhirurgiya.

[16] Burri H (2015). Overcoming the challenge of venous occlusion for lead implantation.. Indian Pacing Electrophysiol J.

[17] Lickfett L., Bitzen A., Arepally A., Nasir K., Wolpert C., Jeong K.M., Krause U., Schimpf R., Lewalter T., Calkins H., Jung W., Lüderitz B. (2004). Incidence of venous obstruction following insertion of an implantable cardioverter defibrillator. A study of systematic contrast venography on patients presenting for their first elective ICD generator replacement. Europace.

[18] Golian M., Vo M., Ravandi A., Seifer C.M (2016). Venoplasty of a chronic venous occlusion allowing for cardiac device lead placement: a team approach.. Indian Pacing Electrophysiol J.

[19] Witte O.A., Adiyaman A., van Bemmel M.W., Smit J.J.J., Ghani A., Misier A.R.R., Elvan A., Delnoy P.P.H.M. (2018). Mechanical power sheath mediated recanalization and lead implantation in patients with venous occlusion: technique and results. J Cardiovasc Electrophysiol.

[20] Bontempi L., Vassanelli F., Cerini M., Inama L., Mitacchione G., Giacopelli D (2018). Video-assisted thoracoscopic monitoring of laser lead extraction by femoral route.. Innovations (Phila).

[21] Goyal S.K., Ellis C.R., Ball S.K., Ahmad R., Hoff S.J., Whalen S.P., Rottman J (2014). High-risk lead removal by planned sequential transvenous laser extraction and minimally invasive right thoracotomy.. J Cardiovasc Electrophysiol.

[22] Migliore F., Cavalli G., Bottio T., Testolina M., De Lazzari M., Bertaglia E., Iliceto S., Gerosa G. (2018). Hybrid minimally invasive technique with the bidirectional rotational Evolution^®^ mechanical sheath for transvenous lead extraction: a collaboration between electrophysiologists and cardiac surgeons. J Arrhythm.

[23] Kiuchi M.G., Andrade R.L.L., Silva G.R.D., Souto H.B., Chen S., Junior H.V (2015). ICD leads extraction and clearing of access way in a patient with superior vena cava syndrome: building a tunnel.. Medicine (Baltimore).

